# A Review of Adherence and Predictors of Adherence to the CONSORT Statement in the Reporting of Tuberculosis Vaccine Trials

**DOI:** 10.3390/vaccines8040770

**Published:** 2020-12-16

**Authors:** Veranyuy D. Ngah, Akhona V. Mazingisa, Moleen Zunza, Charles S. Wiysonge

**Affiliations:** 1 Division of Epidemiology and Biostatistics, Department of Global Health, Faculty of Medicine and Health Sciences, Stellenbosch University, Francie van Zijl Drive, Tygerberg, 7505 Cape Town, South Africa; Moleenz@sun.ac.za (M.Z.); Charles.Wiysonge@mrc.ac.za (C.S.W.); 2 Cochrane South Africa, South African Medical Research Council, Francie van Zijl Drive, Parow Valley, 7501 Cape Town, South Africa; aicomazz@gmail.com; 3 School of Public Health and Family Medicine, University of Cape Town, Anzio Road, Observatory, 7925 Cape Town, South Africa

**Keywords:** tuberculosis, CONSORT, randomized trials, vaccine trials, reporting quality

## Abstract

The statement on Consolidated Standards of Reporting Trials (CONSORT) ensures transparency in the reporting of randomized trials. However, it is unclear if the statement has led to improvement in the quality of reporting of tuberculosis (TB) vaccine trials. We explored the quality of reporting of TB vaccine trials according to the latest version of the CONSORT statement, released in 2010. We searched PubMed and the Cochrane Central Register of Controlled Trials in August 2019. We conducted screening, study selection, and data extraction in duplicate; and resolved differences through discussion. We assessed reporting to be adequate if trials reported at least 75% of the CONSORT 2010 items. We conducted a trend analysis to assess if there was improvement in reporting over time. We also used logistic regression to assess factors associated with adequate reporting. We included 124 trials in the analyses. The mean proportion of adherence was 67.3% (95% confidence interval 64.4% to 70.1%), with only 46 (37%) trials having adequate reporting. There was a significant improvement in the quality of reporting over time (*p* < 0.0001). Trials published in journals with impact factors between 10 and 20 were more likely to have adequate reporting (odds ratio 9.4; 95% confidence interval 1.30 to 67.8), compared to lower-impact-factor journals. Despite advances over time, the reporting of TB vaccine trials is still inadequate and requires improvement.

## 1. Introduction

Tuberculosis (TB) is a bacterial infectious disease which affects mainly the lungs (pulmonary tuberculosis), but also affects other organs (extra-pulmonary tuberculosis). According to the global TB report of 2020, TB features among the top 10 causes of death globally [1]. The Bacille Calmette-Guerin (BCG) vaccine was the first vaccine for TB [2] and it is still the only licensed vaccine for the prevention of the disease [1]. Although it is efficient in providing immunity against extra-pulmonary TB, its protection against pulmonary TB has been shown to vary considerably among different individuals and it is sometimes ineffective [3,4]. Although TB is a treatable disease, the *Mycobacterium tuberculosis* bacteria have become resistant to treatment over the years causing drug-resistant TB, including multidrug-resistant TB and extensively drug-resistant TB [5,6]. This causes a major public health problem and slows down progress made towards control of TB.

In ranking clinical research, randomized trials are rated to provide evidence that is more definitive compared to observational studies. They are the design of choice when conducting medical studies in which treatment interventions are being compared [7]. Due to concerns on the inadequate reporting of trials in the 1990s, the Consolidated Standards for Reporting Trials (CONSORT) statement was developed in 1996 to enhance the quality of trial reporting [8]. CONSORT is a statement consisting of a checklist, a flow diagram, and narrative text. It has been revised and updated twice, with the latest revision occurring in 2010 [9,10].

CONSORT has been used to assess the quality of reporting of randomized trials. In an assessment of the compliance with the CONSORT statement in the reporting head and neck surgery trials in the top 10 Otolaryngology journals, 182 eligible studies were included in the analysis. Adherence to the CONSORT ranged from 25.0% to 93.5% with a mean score of 59.0% [11]. The study revealed that the method of randomization (6.5%), external validity (32.4%), and sample size calculations (40.6%) were among the areas with the greatest insufficiencies in reporting. A similar study assessing the reporting quality of trials in the top 10 journals of critical care medicine reported a median score of 61.1%, with scores of reported items ranging from 33.3% to 86.5%. Items least reported in the studies were changes to methods after the start of the trial (0.0%), modifications of outcomes after the trial commences (0.8%), and methods used in supplementary analyses (7.6%) [12]. The two studies described above were prone to selection bias as the authors limited their search only to top-quality journals. This selection method also limits the generalizability of their results to trials in the specific fields covered by the publications.

Certain factors have been associated with better adherence to the CONSORT. A study to assess adherence and factors influencing adherence to CONSORT in medical oncology showed that journal citation impact factor, recent publication, and geographic location of studies were independent predictors of high reporting quality [13]. Similarly, a study evaluating the reporting of trials of sodium glucose co-transporter 2 inhibitors (an anti-diabetic drug) showed that the journal of publication and the continent where the trial was conducted were independently associated with the quality of reporting of trials [14]. The use of only parallel group design trials in this study limited the sample size of the study which limits the generalizability of the study findings.

A mapping of trials included in Cochrane reviews published from 2011 to 2014 revealed that the use of CONSORT has led to an overall improvement in reporting over the years [15]. However, the examples above show that the reporting of trials in specific diseases and in specific journals is still incomplete and not of utmost standards. Poor reporting of trials can compromise the analysis of a systematic review and the clinical recommendations made [16]. It makes the trial irreproducible, causing a waste of resources [17]. The quality of reporting is therefore crucial for better clarity and transparency in the assessment of study results.

We are not aware of a previous study that has assessed the quality of reporting TB vaccine trials using the CONSORT statement. Given that TB is a global health problem and that vaccines are an important way of reducing morbidity and mortality, it is important to assess the quality of reporting of TB vaccine trials. We assessed the adherence to the CONSORT 2010 statement of TB vaccine trials published from 1990 to 2018. We hypothesized that studies published after the release of the CONSORT statement, journal endorsement of CONSORT, non-industrial funding, high impact factor, and geography would be associated with adequate reporting.

## 2. Materials and Methods

### 2.1. Study Design

We conducted a review of the reporting of TB vaccine trials with no restriction to study type (i.e., superiority, non-inferiority, or equivalence trials). Trials were eligible if they were published between 1990 and 2018, irrespective of the location where they were conducted. Non-human studies and studies not published as full papers (such as conference abstracts) were excluded as they are unlikely to report all CONSORT items.

### 2.2. Search and Selection of Studies

With the assistance of the Stellenbosch University health science librarian, we developed and implemented a comprehensive search in PubMed and the Cochrane Central Register of Controlled Trials (CENTRAL) on the on the 21st of August 2019. We restricted the search to human studies and to studies published between January 1990 and December 2018. The details of the search strategy are shown in Table 1. One researcher (VN) screened the titles and abstracts and excluded irrelevant studies from the list; after duplicates were removed using study title, authors, and DOI numbers. Two studies (VN and AM) then independently assessed the full text of potentially eligible studies, as well as selected studies that met inclusion criteria. Data extraction was conducted independently in duplicate (VN and AM) using a form adapted from the CONSORT checklist (Appendix A). Discrepancies were discussed and resolved by consultation with a third researcher (CI). The Cohen’s K statistics [18] for inter-observer agreement was 0.76. All researchers were trained on research methods and the CONSORT checklist.

### 2.3. Assessment of Reporting Quality

The assessment was conducted in duplicate by two independent researchers (VN and AM) to minimize reviewer subjective bias. The CONSORT checklist has 37 items. Item 13c (a flow diagram) was included as its importance is stressed in the CONSORT explanation and elaborations. The use of the checklist was piloted on three randomly selected studies by two researchers (VN and AM). Prior to the data collection, we discussed the meaning and interpretations of the items on the CONSORT list and how to judge if the item was reported, unclear or not reported based on the explanations provided in the CONSORT guidance. A score of 1 was given if an item was reported and 0 if not reported or if reporting was unclear. Items reported in the supplementary materials of trials were scored as reported when reference was mentioned in the main article, apart from item 8a as it must be mentioned in the main article according to the CONSORT explanations and elaborations. Some items were non-applicable due to the design of the study. We calculated the proportion of the items reported for each study as “the number of CONSORT items reported” divided by “the total number of CONSORT items applicable to the study”.

### 2.4. Explanatory Variables

#### 2.4.1. Journal Endorsement of CONSORT

This was classified into CONSORT endorsing journals (that is, journals requesting authors to report according to the CONSORT statement) and non-CONSORT endorsing journals.

#### 2.4.2. Funding Type

Funding was categorized as industrial funding, non-industrial funding, and no funding. Industrial funding included funding from pharmaceutical companies, funding from organizations conducting TB research, and any other sources of private funding. Funding classified as non-industrial was from the government and governmental institutions. We assumed that if funding was not mentioned, the study was not funded. Thus, “no funding” was used when funding was not mentioned or when it was mentioned that the study was not funded.

#### 2.4.3. Journal Impact Factor

The studies were divided into three categories: impact factor less than 10, impact factor greater than 10 but less than 20, and impact factor greater than 20. The journal impact factors used were those of the year when the study was published. A journal impact factor of 10 and above is considered high in most study fields [19]. The journal impact factor was accessed through the university of Stellenbosch access to the Journal Citation Reports on the Web of Science group database.

#### 2.4.4. Continent Where the Study Was Conducted

This was the continent from which study participants were recruited. This was divided into the six continents: (1) Africa, (2) North America, (3) South America, (4) Europe, (5) Asia, and (6) Australia and New Zealand.

#### 2.4.5. Other Exploratory Variables

We included the year of publication and the number of study centers.

### 2.5. Outcome Variable

Although the CONSORT statement does not specify what percentage of reported items should be considered adequate reporting, we defined adequate reporting using the definition used in previous studies [20,21]. We considered reporting to be adequate in each trial if the trial reported at least 75% of the items on the CONSORT 2010 checklist applicable to the trial.

### 2.6. Sample Size Estimation

The number of articles that would have adequate reporting of the CONSORT items was used for sample size estimation. Assuming that 57% of the studies would have adequate reporting based on a previous study [21], according to a priori sample size determination, a sample size of 105 was required to get a precision of ±10% and a sample size of 128 would give precision of ±9%. In our study, we obtained a sample of 124 studies.

### 2.7. Data Analysis

We used STATA statistical software version 16.0 for data analysis (StataCorp, Collage Station, TX, USA). We summarized categorical variables as count (percent) and continuous variables as mean (standard deviation). A trend analysis was conducted to assess if there was improvement in reporting quality over time. We used logistic regression to assess factors associated with adequate reporting. Characteristics with a *p*-value < 0.1 in bivariate analyses were entered into a multiple logistic regression model to determine factors associated with adequate reporting after adjustment for potential confounding. In the bivariate logistic regression analyses, we assessed the association between each exploratory variable and the outcome (i.e., adequate reporting). A significance level of 0.1 was considered as a conservative screen for identifying potential factors associated with adequate reporting and thus minimize the number of factors that might be excluded in the bivariate analyses. We report the odds ratio (OR) as a measure of association, with the corresponding 95% confidence interval (CI). Statistical significance was set at a *p*-value less than 0.05 in the multiple logistic regression model.

### 2.8. Ethical Considerations and Reporting

The population of study was published TB vaccine trials, which are publicly available. Therefore, the study was exempt from ethics approval. The study did not involve direct participation of human individuals, and as such was of minimal risk and informed consent was not required. The study was reported in accordance with the preferred reporting items for systematic reviews and meta-analyses (PRISMA) statement [22].

## 3. Results

### 3.1. Study Search and Selection

**Figure 1:**
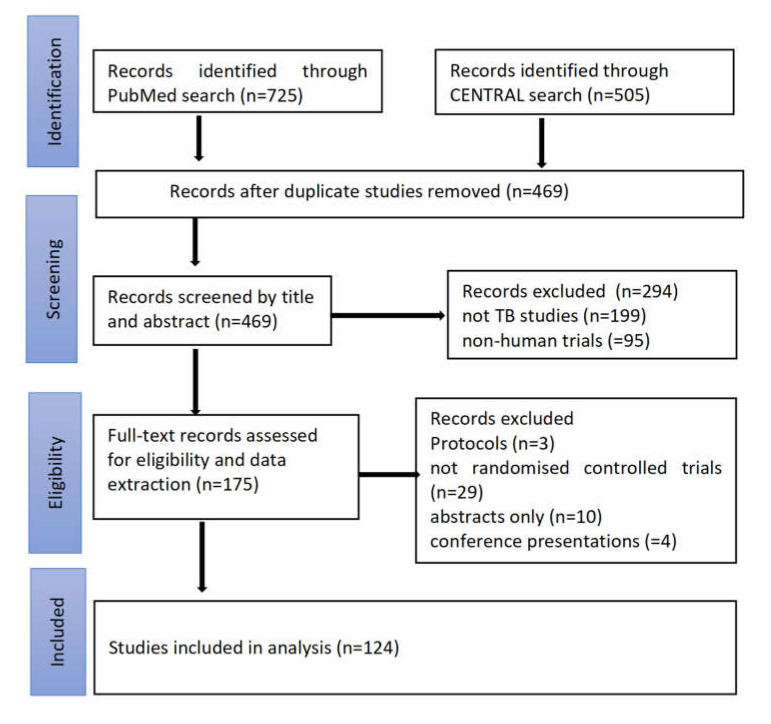
Study flow diagram showing the search and selection process for the study.

A total of 1230 reports were obtained from both the PubMed and CENTRAL databases, 469 reports were retained after the removal of duplicates in Mendeley, 294 reports were excluded after screening the titles and abstracts, and 51 were excluded after screening the full texts (Figure 1). A total of 124 trials were eligible for data extraction and analysis. These studies were published in six continents; 56 (45%) were published in Africa, 35 (38%) in Europe, 13 (10%) in Asia, 10 (8%) in North America, 8 (7%) in South America, and 2 (2%) in Australia.

### 3.2. Overall Quality of Reporting

The mean proportion of adherence was 67.3% (95% CI 64.4% to 70.1%), the standard deviation was 16.2%, and the range was from 26.5% to 97.1%. Forty-six studies (37%) adequately reported the items of the CONSORT checklist (i.e., reported at least 75% of the items).

**Figure 2:**
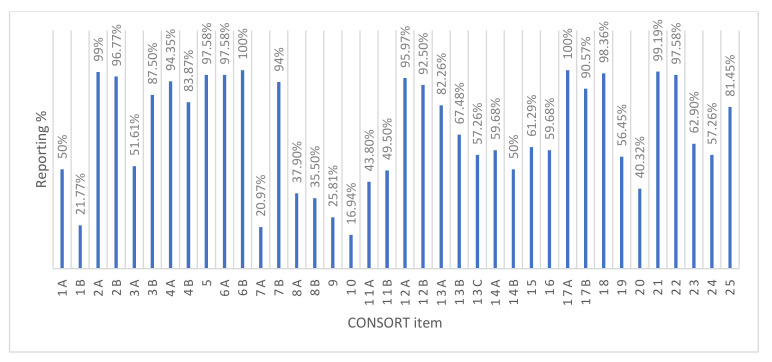
Percentage of each Consolidated Standards of Reporting Trials (CONSORT) item reported.

Figure 2 shows the proportion of each reported CONSORT item by the 124 studies. The CONSORT items are explained in Appendix A (Data extraction form Part 2: CONSORT items). The least reported methodological items were: who generated the random allocation sequence and assigned participants (16.9%), how the random allocation sequence was generated (37.9%), the type of randomization (35.5%), the implementation of random allocation sequence (26%), planned interim analysis (13.7%), and sample size determination (21%). The items adequately reported were scientific background and rationale (99.2%), specific objectives (96.8%), eligibility criteria (94.4%), pre-specified primary and secondary outcomes (97.6%), statistical methods (96%), results of primary and secondary outcomes (100%), and source of funding (81.5%).

### 3.3. Evolution in Reporting Quality

There was a significant improvement in the quality of reporting over time (*p* < 0.001); 4.4% of studies published between 1990 and 2000 had adequate reporting, 21.4% of the studies published between 2001 and 2010 had adequate reporting, and 53.4% of studies published between 2011 and 2018 had adequate reporting.

### 3.4. Factors Associated with Adequate Reporting

The odds of adequate reporting for studies published in journals with impact factors between 10 and 20 were nine times the odds of studies in journals with impact factors less than 10 (OR 9.4 95% CI 1.30 to 67.8) (Table 2). The odds of adequate reporting also increased with the year of publication (*p* < 0.01). Journal endorsement of the CONSORT statement had a 56% non-significant increase in the odds of adequate reporting.

## 4. Discussion

We found that the quality of reporting of TB vaccine trials improved significantly with time. This is in line with several studies that have reported improvement of trial reporting quality over time [20,22,23]. This could be because authors and editors are becoming more aware of the importance of adequate reporting and the importance of the CONSORT guideline in reporting trials [24].

Although there was improvement in the reporting quality of TB vaccine trials over time, the overall reporting quality was assessed as inadequate as only 37% of the studies had adequate reporting. Our findings are similar to those reported in a study evaluating the reporting of trials in endodontic journals. A mean score of 66% was reported, and it was concluded that, although reporting improved over time, the overall quality of reporting was not adequate [23]. Several other studies conducted in various medical fields have also demonstrated sub-optimal reporting according to CONSORT [20,24,25,26].

The reporting of several methodological items was generally poor. The identification of a study as a randomized trial in the title helps database indexers to be able to index the study correctly in electronic databases. This eases the online search for trials and prevents studies from being missed in a targeted search for trials. In this study, up to 78.2% of the studies were not identified as randomized trials in the title. In addition, sample size determination was one of the least reported items. Poor reporting of sample size determination has also been reported by other authors [25,27,28]. The calculation of sample size a priori provides evidence that the study is powered enough to validate any statistically significant difference observed between the intervention and control groups. It also prevents the wastage of resources (in over-powered studies) and the needless subjection of participants to possible harmful interventions.

The generation of random allocation sequence, the type of randomization, implementation of the random allocation sequence and who generated the allocation sequence are all part of the randomization process in trials and were poorly reported. Poor reporting of these items has been shown by other authors [29]. Inadequate information on the randomization process provides room for doubt in the validity of the study. It causes uncertainty of the randomization process and whether the sequence was tampered with, introducing selection bias that could lead to an exaggeration of the treatment effect [30].

Although most of the trials described their studies as blinded and double blinded, the stating of who was blinded after assignment of the intervention was described only by 43.8% of the studies. In examining the reporting quality of trials in chiropractic practice, only 46% of the studies published between 2005 and 2014 reported on blinding [31]. Blinding reduces measurement bias which can be introduced intentionally or unintentionally as observer bias especially in patient-reported outcomes [32].

Intention-to-treat analysis was reported only in about 60% of the studies. This method of analysis is usually favored over per-protocol analysis. It reduces the chances of attrition bias that could arise from non-random loss of study participants that occur from loss-to-follow-up of study participants [33,34] Very poor reporting of intention-to-treat analysis (12%) in trials of scalp acupuncture treatment for vascular dementia has also been reported [27].

Some reasons could explain the poor reporting of these items. One reason could be that these studies are poorly conducted as the quality of reporting has been shown to be related to methodological quality [35]. However, we have also seen that studies that have been properly conducted with high methodological quality can be poorly reported [36]. Therefore, poor reporting is not always a reflection of poor methodological quality.

Another reason could be that studies published before 2010 could be using the old version of the CONSORT statement published in 1996 [8]. However, even so, they should have placed more emphasis on the methodological items that are listed as this would increase the validity of their reported studies. There is also the possibility of word limitation imposed by the journal editors; however, editors could then provide ways for other information to be made available such as online supplementary material, hence the main article contains the important methodological aspects.

The type of funding was not associated with the quality of reporting. Similar findings have been reported by others [31]. This is in contrast with other findings [37,38]. While one suggested that industrial funding caused researchers to be more meticulous in their methodology [37], the other concluded that industrial funding could lead to publication bias such that only well-conducted studies with positive outcomes are published [38].

Journal endorsement of CONSORT was not associated with adequate reporting. In the evaluation of the quality of reporting in trials for novel oral anticoagulants in venous thromboembolic disease, similar findings were reported [39]. Contrasting findings have been reported where journals endorsing CONSORT had studies with better reporting quality than non-endorsing journals [20,40]. The association of CONSORT endorsement with better reporting quality could be confounded by citation impact factors [40].

The number of study centers was not associated with the quality of reporting. Other authors have also found no significant association between the number of study sites and the quality of CONSORT item reporting [41]. However, another study reported that multicenter studies were statistically associated with a higher quality of reporting of surgery trials [42].

Studies published in journals with impact factors between 10 and 20 were significantly associated with adequate reporting but studies published in journals with impact factor above 20 were not significantly associated with adequate reporting. Journals with high impact factors are more compliant to the CONSORT as adherence to the CONSORT is a requirement for these journals. A systematic review on the influence of impact factor on the methodological quality of surgical trials published in journals with high impact factors was associated with improved methodological quality. However, the impact factors in this study were only classified as less than 2 or more than 3. Moreover, impact factor in this study was not independent of industrial funding and positive results outcome, which could both have been confounding factors [43].

The continent of study was not significantly associated with the quality of reporting. In contrast to our findings, some studies have reported that studies conducted outside of North America have a higher quality of reporting [13,14].

## 5. Strengths and Limitations

A systematic search was applied to obtain all human TB vaccine trials with no limitations except for human trials and the years of interest. To minimize selection bias, two researchers independently conducted study selection and data extraction. Discrepancies between the researchers in the study selection and data extraction were resolved by input from a third researcher.

This study was evaluating the reporting quality of TB vaccine trials published as full papers. Abstracts, conference papers and unpublished studies were excluded from the study rendering the study vulnerable to publication bias.

The scoring process used in this study placed equal weight on all the items of the CONSORT list. We understand that some parts of the CONSORT such as the methodology have been given more importance. As such, two studies may have the same score, but one may be considered more deficient if it has poorer reporting of its methodology.

This study was an assessment of the reporting quality of TB vaccine trials and not an assessment of the methodological quality or if the studies reported were properly conducted. The results of the various studies were not being analyzed, nor were they being assessed for bias. As such, a study that could be bias but was well reported was given full credit for its reporting.

## 6. Conclusions and Recommendations

Our study observed that, although the overall quality of reporting in TB vaccine trials was inadequate, there was improvement in the quality of reporting with time. These findings do not discredit the evidence in these studies but serve to enlighten the TB vaccine research community that more emphasis is needed for appropriate and adequate reporting of studies. This is especially important as there are still several on-going TB vaccine studies that rely on the evidence being put out from previous studies. We recommend that journal editors should be more stringent in recommending that authors should submit a completed CONSORT checklist together with their manuscripts for publication. We encourage authors to report their methodology in a better manner, especially sample size determination and the process of randomization and blinding.

## Figures and Tables

**Table 1:** Search strategy used in the study.

A. Search String Used for the PubMed Database	
#	Search
1	Tuberculosis OR TB OR PTB
2	Vaccine OR Vaccines OR Vaccination
3	Placebo OR control
4	“randomized controlled trials” OR randomization OR RCT OR controlled trials OR Comparative
5	(((Tuberculosis OR TB OR PTB) AND (Vaccine OR Vaccines OR Vaccination)) AND (Placebo OR control)) AND (“randomized controlled trials” OR randomization OR RCT OR controlled trials OR Comparative)
6	(((Tuberculosis OR TB OR PTB) AND (Vaccine OR Vaccines OR Vaccination)) AND (Placebo OR control)) AND (“randomized controlled trials” OR randomization OR RCT OR controlled trials OR Comparative) Filters: Humans
7	(((Tuberculosis OR TB OR PTB) AND (Vaccine OR Vaccines OR Vaccination)) AND (Placebo OR control)) AND (“randomized controlled trials” OR randomization OR RCT OR controlled trials OR Comparative) Filters: Humans, from 1990–2020
8	(((Tuberculosis OR TB OR PTB) AND (Vaccine OR Vaccines OR Vaccination)) AND (Placebo OR control)) AND (“randomized controlled trials” OR randomization OR RCT OR controlled trials OR Comparative) Filters: Humans, from 1990–2018
**B. Search String Used for the Cochrane Central Register of Controlled Trials**	
**#**	**Search**
1	MeSH descriptor: [Tuberculosis] explode all tress
2	MeSH descriptor: [Vaccines] explode all tress
3	MeSH descriptor: [Vaccination] explode all tress
4	#2 OR #3
5	#1 AND #4

**Table 2:** Study characteristics associated with adequate reporting.

Characteristic	Categories Used for the Characteristic	Crude Odds Ratio * (95% CI)	*p* Value	Adjusted Odds Ratio ** (95% CI)	*p* Value
Impact factor	<10	Reference		Reference	
	10 to 20	6.44 (1.24 to 33.58)	0.027	9.4 (1.30 to 67.83)	0.026
	>20	4.29 (1.01 to 18.20)	0.048	2.89 (0.30 to 27.80)	0.35
Type of Funding	Industrial funding	Reference		Reference	
	Non-industrial funding	0.28 (0.12 to 0.65)	0.003	0.55 (0.18 to 1.67)	0.29
	No funding	0.09 (0.023 to 0.33)	<0.001	0.23 (0.45 to 1.26)	0.09
Type of journal	Non-CONSORT endorsing	Reference		Reference	
	CONSORT endorsing	2.33 (1.04 to 5.26)	0.041	1.85 (0.59 to 5.84)	0.28
Year of publication	1990–2000	Reference		Reference	
	2001–2010	5.99 (0.67 to 54.04)	0.110	4.89 (0.38 to 62.45)	0.22
	2011–2018	25.24 (3.23 to 197.22)	0.002	25.8 (2.25 to 297.20)	0.009
Continent of study	Africa	Reference		Reference	
	Asia	0.44 (0.12 to 1.61)	0.218	1.45 (0.22 to 9.56)	0.69
	Europe	0.52 (0.22 to 1.25)	0.144	0.65 (0.22 to 1.93)	0.44
	North America				
	South America	1 (0.059 to 16.79)	1.000	0.24 (0.007 to 8.40)	0.43
Number of centers	Single center	Reference			
	Multiple centers	1.344 (0.64 to 2.81)	0.432		

CI, confidence interval. The crude odds ratios (*) show the results from the bivariate logistic regression analyses, and adjusted odds ratios (**) show results from the multiple logistic regression analysis. The latter was performed on all variables in the model.

## Appendix A. Data Extraction Form

**Table A1:** Part 1: General Information.

Study Number				
Journal Endorsement of CONSORT	Yes		No	
Journal impact factor	IF < 10	10 < IF < 20		IF > 20
Continent of trial				
Number of trial centers	Single center		multicenter	
Source of funding	Industrial	Non-industrial		No funding
Year of publication	1990–2000	2001–2010		2011–2018

**Table A2:** Part 2: CONSORT Items.

Section	Item No.	Checklist Item	Yes	No	Score
Title and Abstract	1a	Identification as a randomized trial in the title			
	1b	Structured summary of trial design, methods, results, and conclusions			
Background and objectives	2a	Scientific background and explanation of rationale			
	2b	Specific objectives or hypotheses			
Trial design	3a	Description of trial design (such as parallel, factorial) including allocation ratio			
	3b	Important changes to methods after trial commencement (such as eligibility criteria), with reasons			
Participants	4a	Eligibility criteria for participants			
	4b	Settings and locations where the data were collected			
Interventions	5	The interventions for each group with sufficient details to allow replication, including how and when they were actually administered			
Outcomes	6a	Completely defined pre-specified primary and secondary outcome measures, including how and when they were assessed			
	6b	Any changes to trial outcomes after the trial commenced, with reasons			
**Section**	**Item No.**	**Checklist Item**	**Yes**	**No**	**Score**
Sample size	7a	How sample size was determined			
	7b	When applicable, explanation of any interim analyses and stopping guidelines			
Sequence generation	8a	Method used to generate the random allocation sequence			
	8b	Type of randomization; details of any restriction (such as blocking and block size)			
Allocation concealment mechanism	9	Mechanism used to implement the random allocation sequence (such as sequentially numbered containers), describing any steps taken to conceal the sequence until interventions were assigned			
Implementation	10	Who generated the random allocation sequence, who enrolled participants, and who assigned participants to interventions			
Blinding	11a	If done, who was blinded after assignment to interventions (for example, participants, care providers, those assessing outcomes) and how			
	11b	If relevant, description of the similarity of interventions			
Statistical methods	12a	Statistical methods used to compare groups for primary and secondary outcomes			
	12b	Methods for additional analyses, such as subgroup analyses and adjusted analyses			
Participant flow	13a	For each group, the numbers of participants who were randomly assigned, received intended treatment, and were analyzed for the primary outcome			
	13b	For each group, losses and exclusions after randomization, together with reasons			
	13c	Flow diagram			
Recruitment	14a	Dates defining the periods of recruitment and follow-up			
	14b	Why the trial ended or was stopped			
Baseline data	15	A table showing baseline demographic and clinical characteristics for each group			
Numbers analysed	16	For each group, number of participants (denominator) included in each analysis and whether the analysis was by original assigned groups			
Outcomes and estimation	17a	For each primary and secondary outcome, results for each group, and the estimated effect size and its precision (such as 95% confidence interval)			
	17b	For binary outcomes, presentation of both absolute and relative effect sizes is recommended			
Ancillary analyses	18	Results of any other analyses performed, including subgroup analyses and adjusted analyses, distinguishing pre-specified from exploratory			
Harms	19	All-important harms or unintended effects in each group			
Limitations	20	Trial limitations, addressing sources of potential bias, imprecision, and, if relevant, multiplicity of analyses			
Generalizability	21	Generalizability of the trial findings			
Interpretation	22	Interpretation consistent with results, balancing benefits and harms, and considering other relevant evidence			
Registration	23	Registration number and name of trial registry			
Protocol	24	Where the full trial protocol can be accessed, if available			
Funding	25	Sources of funding and other support role of funders			
